# Profiling of Substrate Specificity of SARS-CoV 3CL^pro^

**DOI:** 10.1371/journal.pone.0013197

**Published:** 2010-10-06

**Authors:** Chi-Pang Chuck, Lin-Tat Chong, Chao Chen, Hak-Fun Chow, David Chi-Cheong Wan, Kam-Bo Wong

**Affiliations:** 1 School of Biochemical Sciences, The Chinese University of Hong Kong, Sha Tin, Hong Kong, China; 2 Department of Biochemistry and Molecular Biotechnology Programme, The Chinese University of Hong Kong, Sha Tin, Hong Kong, China; 3 Department of Chemistry, The Chinese University of Hong Kong, Sha Tin, Hong Kong, China; Griffith University, Australia

## Abstract

**Background:**

The 3C-like protease (3CL^pro^) of severe acute respiratory syndrome-coronavirus is required for autoprocessing of the polyprotein, and is a potential target for treating coronaviral infection.

**Methodology/Principal Findings:**

To obtain a thorough understanding of substrate specificity of the protease, a substrate library of 19
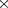
8 variants was created by performing saturation mutagenesis on the autocleavage sequence at P5 to P3' positions. The substrate sequences were inserted between cyan and yellow fluorescent proteins so that the cleavage rates were monitored by *in vitro* fluorescence resonance energy transfer. The relative cleavage rate for different substrate sequences was correlated with various structural properties. P5 and P3 positions prefer residues with high β-sheet propensity; P4 prefers small hydrophobic residues; P2 prefers hydrophobic residues without β-branch. Gln is the best residue at P1 position, but observable cleavage can be detected with His and Met substitutions. P1' position prefers small residues, while P2' and P3' positions have no strong preference on residue substitutions. Noteworthy, solvent exposed sites such as P5, P3 and P3' positions favour positively charged residues over negatively charged one, suggesting that electrostatic interactions may play a role in catalysis. A super-active substrate, which combined the preferred residues at P5 to P1 positions, was found to have 2.8 fold higher activity than the wild-type sequence.

**Conclusions/Significance:**

Our results demonstrated a strong structure-activity relationship between the 3CL^pro^ and its substrate. The substrate specificity profiled in this study may provide insights into a rational design of peptidomimetic inhibitors.

## Introduction

Severe acute respiratory syndrome-coronavirus (SARS-CoV) is the causative agent of a lethal pneumonia discovered in 2003 [1], [2]. The single-stranded RNA viral genome encodes two polyproteins consisting of 15 non-structural proteins [3], [4]. Activation of these non-structural proteins requires proteolytic cleavage by papain-like protease and 3C-like protease (3CL^pro^). Inhibiting 3CL^pro^ proteolysis is a convincing strategy against SARS because it suppresses viral replication and virus-induced cytopathic effects [5], [6], [7], [8].

Native 3CL^pro^ is a homodimer. Each protomer of 34 kDa is divided into three domains [9], [10], [11]. Domain I (residue 8–101) and II (residue 102–184) form a substrate-binding cleft, while domain III (residue 201–303) is responsible for dimerization. Catalytic mechanism of 3CL^pro^ resembles that of a typical cysteine protease. Cleavage of the peptide bond between P1 and P1' positions is catalyzed by the Cys145 and His41 dyad [12]. Domain III is also essential in the proteolysis, as the protease is active only in dimeric conformation [13].

Gln is absolutely conserved at P1 position among the 11 3CL^pro^ cleavage sites in the polyproteins. Previous studies showed that P2 position accommodates hydrophobic residues with large side chains such as Leu and Phe, while P1' position tolerates small-sized residues [14], [15]. Substitutions at P5 to P3' positions were found to affect the 3CL^pro^ activity, but comprehensive studies on substrate specificity at these positions are scarce [16], [17].

Chu *et al.* synthesized peptide substrates with single residue substitution at each of the P4, P3, P2, P1', P2' and P3' positions [18]. The cleavage of these peptide substrates by 3CL^pro^ was detected by mass spectrometry. They showed that cleavage was detected only when Leu and Phe were present at P2 position. Peptide substrates with acidic residues at P1' position and with Ile/Leu at P2' position were not cleavable. All substitutions at P4 and P3' positions resulted in cleavable substrates. Their detection method can only determine qualitatively whether the peptide substrate is cleavable, but not the relative cleavage rate of different substitutions. Goetz *et al.* profiled the specificity at P4 to P1 positions using a fully degenerate library of tetrapeptides linked with a fluorogenic group at the C-terminus [19]. Contradictory to the common belief that P1 position only takes Gln, they showed that 3CL^pro^ can cleave the peptide substrates containing His at P1 position equally well. However, in their hands, peptide substrates with Phe at P2 position have no observable cleavage, which is inconsistent with the observation that Phe is naturally occurring at this position of the autocleavage sequence of polyproteins. It is, therefore, unknown whether the tetrapeptide is a good model for substrate specificity for 3CL^pro^.

Here, we report the substrate specificity of SARS-CoV 3CL^pro^ at P5 to P3' positions by using protein substrates. The wild-type (WT) protein substrate consists of the autocleavage sequence (TSAVLQ↓SGFRKM) inserted between cyan and yellow fluorescent proteins (CFP and YFP) so that the cleavage can be monitored by fluorescence resonance energy transfer (FRET). We created a substrate library of 19
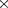
8 variants by saturation mutagenesis at each of P5 to P3' positions, and measured the cleavage rate of 3CL^pro^ against these substrate variants. The results were correlated with various properties of substituting residues including side chain volume, hydrophobicity and α-helix and β-sheet propensities [20], [21], [22]. The substrate specificity of SARS-CoV 3CL^pro^ was discussed based on the quantitative correlation obtained.

## Results and Discussion

### SARS-CoV 3CL^pro^ proteolytic rate was examined by FRET assay

**Figure 1:**
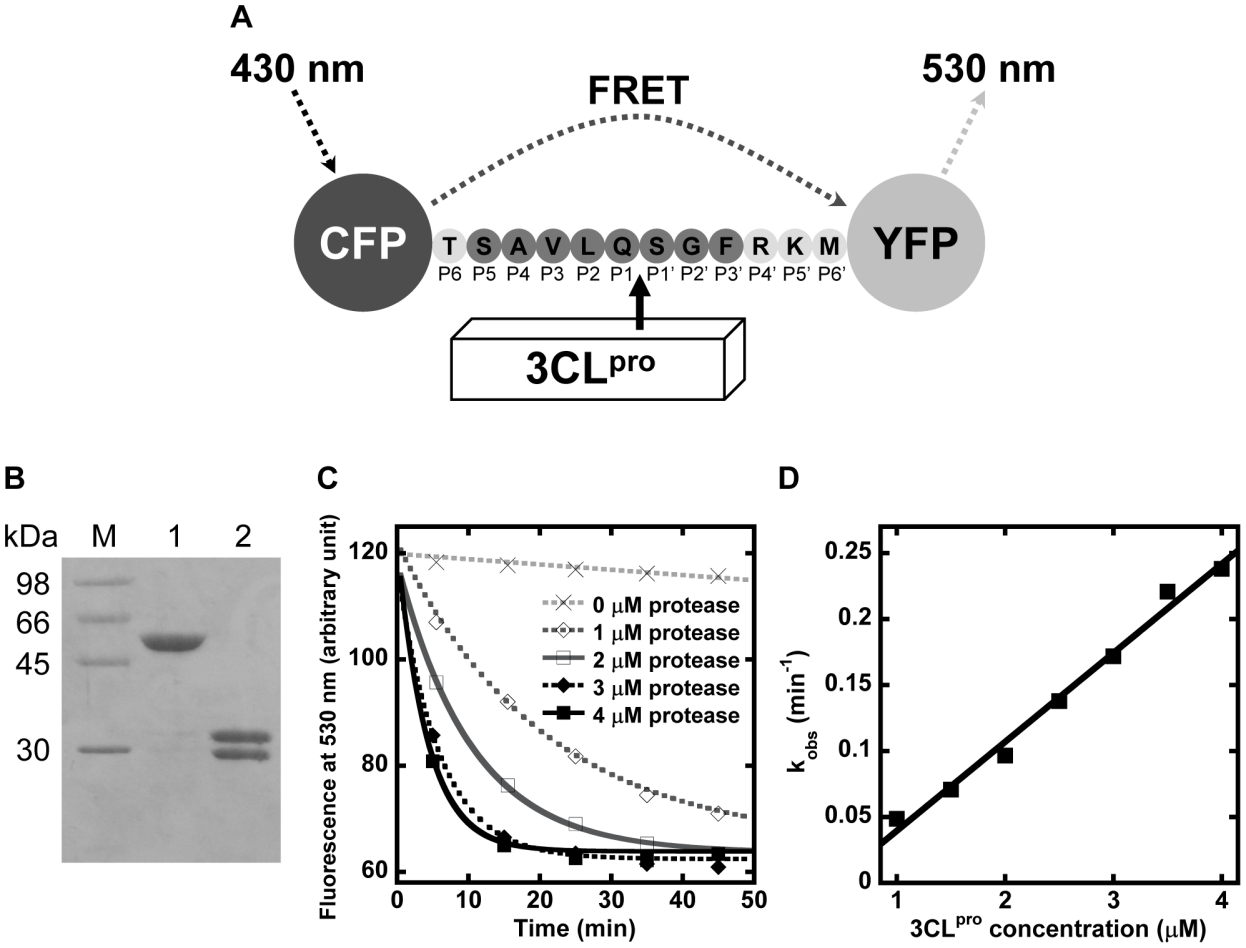
Determination of SARS-CoV 3CL^pro^ proteolytic rate using the protein substrate by FRET assay. (A) Schematic diagram illustrating the principle of the FRET assay. The autocleavage sequence of 3CL^pro^ is inserted between CFP and YFP in the protein substrate. When CFP is excited at 430 nm, YFP emits fluorescence at 530 nm through FRET. Cleavage of the peptide bond at SAVLQ↓SGF by 3CL^pro^ separates CFP and YFP, leading to a decrease in the emitted fluorescence at 530nm. (B) After digestion by 4 µM of 3CL^pro^ for one hour, the protein substrate of 58 kDa (lane 1) was separated into two products of 28 kDa and 30 kDa (lane 2). (C) The protein substrate cleaved by 1 to 4 µM of 3CL^pro^ led to a time-dependent decrease in fluorescence at 530 nm. Observed rate constant, k_obs_, was obtained by fitting the data to a single exponential decay. (D) The plot of k_obs_ against [3CL^pro^] yielded a straight line. The specific activity, k_obs_/[3CL^pro^], was determined by the slope of the plot.

The recombinant protein substrate comprised the autocleavage sequence (TSAVLQ↓SGFRKM) inserted between CFP and YFP (Figure 1A). After digestion by 3CL^pro^, the substrate of 58 kDa was cleaved into two fragments of 28 and 30 kDa (Figure 1B). N-terminal sequencing confirmed that 3CL^pro^ cleaved the protein substrate specifically at the peptide bond between P1 and P1' positions. Separation of the two fluorescent proteins caused the reduction in FRET efficiency, and the reaction rate was followed by time-dependent decrease of emitted fluorescence at 530 nm (Figure 1C). The observed rate constants, k_obs_, were measured at 1 to 4 µM of 3CL^pro^. The specific activity, k_obs_/[3CL^pro^], for WT autocleavage sequence was determined by the slope of k_obs_ against 3CL^pro^, which was 71±11 mM^−1^ min^−1^ (Figure 1D).

### Profiling of substrate specificity at P5 to P3' positions

**Figure 2:**
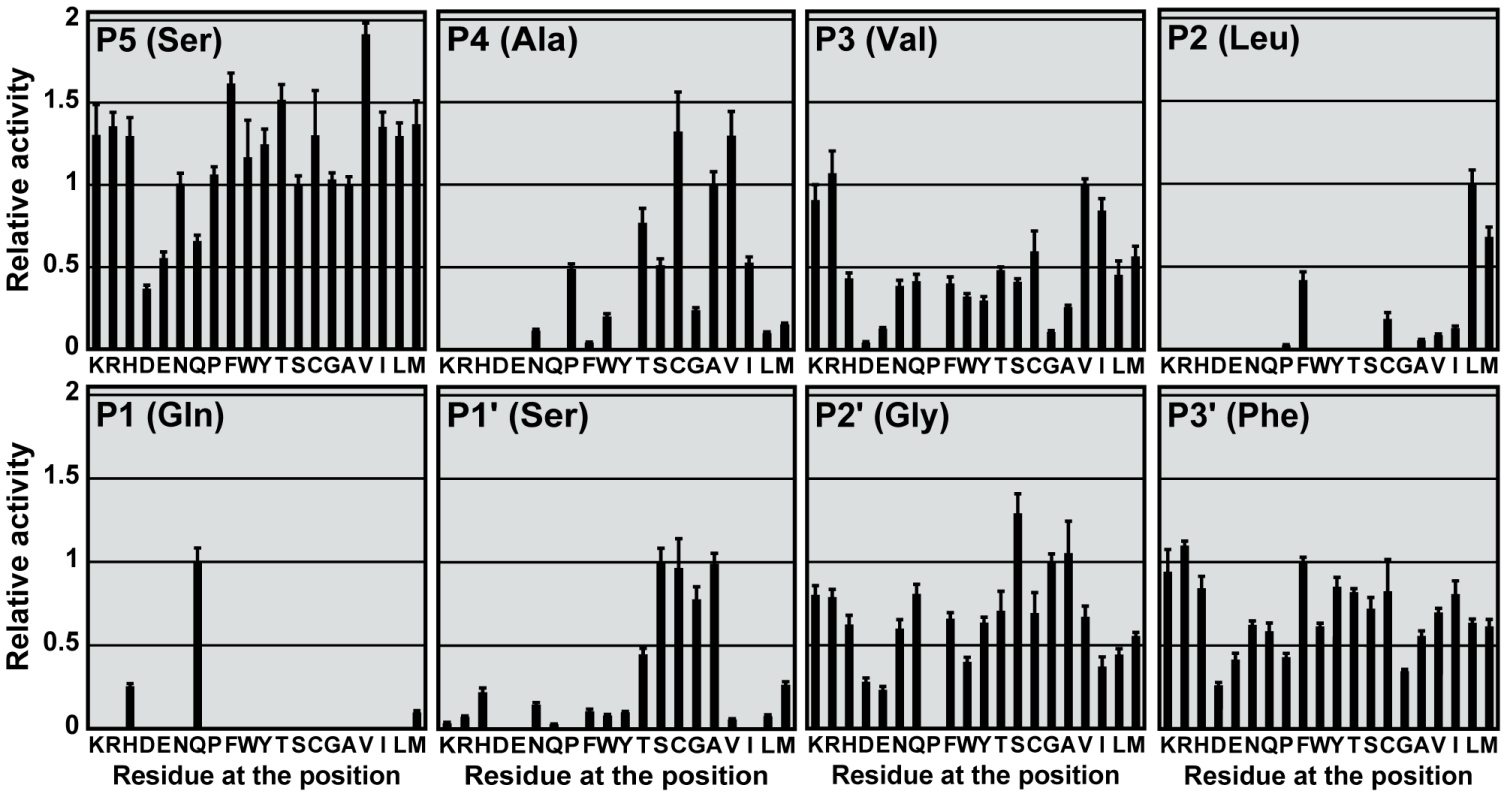
Profiling the substrate specificity at P5 to P3' positions. 19
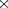
8 single substitution variants were created by saturation mutagenesis of the autocleavage sequence at P5 to P3' positions. Specific activity on each of variants was determined, and normalized by that on WT substrate to obtain the relative activity.

We performed saturation mutagenesis at P5 to P3' positions of the autocleavage sequence to create a substrate library of 19
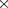
8 variants. The relative cleavage rate of 3CL^pro^ against these substrate sequences was measured (Figure 2, Table S1). In general, solvent-exposed sites such as P5, P3, and P3' positions were less selective than the others. The most selective site was P1 position – cleavage was only observable with Gln, His or Met. Substrate sequences with Pro substitutions at P3, P1', P2' positions were not cleavable.

We also noted that solvent-exposed sites such as P5, P3 and P3' positions preferred positively charged substitutions, as the protease activity on the Arg/Lys-substituting variants was consistently higher than that of the Asp/Glu-substituting variants. The difference was the largest at P3 position, where positively charged substitutions resulted in 12-fold higher in proteolytic activity. P5 and P3' variants with positive charges were also 3-fold higher in activity.

The preference on charged residues indicated that electrostatic interaction, which is long-range in nature, may play a role in 3CL^pro^ catalysis. One of the possibilities is that the positive charges stabilize the transition state of catalysis. It is expected that the carboxylate group at P1 residue will be converted to an oxyanion during the formation of the transition state. Presence of positive charges near the active site may electrostatically stabilize the oxyanion and thus promote catalysis. Another possibility is a direct electrostatic interaction between positively charged residues of substrate and negatively charged residues of 3CL^pro^. There is a Glu166 located at substrate binding cleft that can interact with P3 residue. This may explain why P3 position has the strongest preference for positively charged residues.

To demonstrate the preferred properties of substituting residues, 3CL^pro^ activity was correlated with side chain volume, hydrophobicity, and α-helix and β-sheet propensities [20], [21], [22]. The correlation coefficients (r) and p-values were showed in table 1. Significant correlations with p-value<0.01 were observed in a number of cases and the substrate preferences for each of the positions were discussed below.

**Table 1:** Correlation between SARS-CoV 3CL^pro^ activity and structural properties of substituting residues.

Position	Side chain volume	Hydrophobicity	α-helix propensity	β-sheet propensity
P5	0.331 (0.154)	0.573 (0.008)*	−0.064 (0.789)	0.711 (<0.001)*
P4	−0.424 (0.063)	0.587 (0.006)*	−0.147 (0.536)	0.315 (0.176)
P3	0.338 (0.144)	0.221 (0.349)	0.170 (0.473)	0.510 (0.022)
P2	0.255 (0.277)	0.590 (0.006)*	0.379 (0.100)	0.304 (0.192)
P1	0.038 (0.873)	−0.269 (0.252)	0.126 (0.595)	0.021 (0.931)
P1'	−0.660 (0.002)*	0.233 (0.323)	−0.222 (0.347)	−0.143 (0.548)
P2'	−0.363 (0.116)	0.022 (0.926)	−0.097 (0.685)	0.048 (0.841)
P3'	0.496 (0.026)	0.094 (0.695)	−0.017 (0.944)	0.486 (0.030)

The relative activity was correlated with scales for side chain volume, hydrophobicity, and α-helix and β-sheet propensities of the substituting residues [20], [21], [22]. The correlation coefficients and p-values (in parenthesis) were reported. Significant correlations with p-value<0.01 were bolded and marked with asterisks.

### P5 position prefers residues with high β-sheet propensity

**Figure 3:**
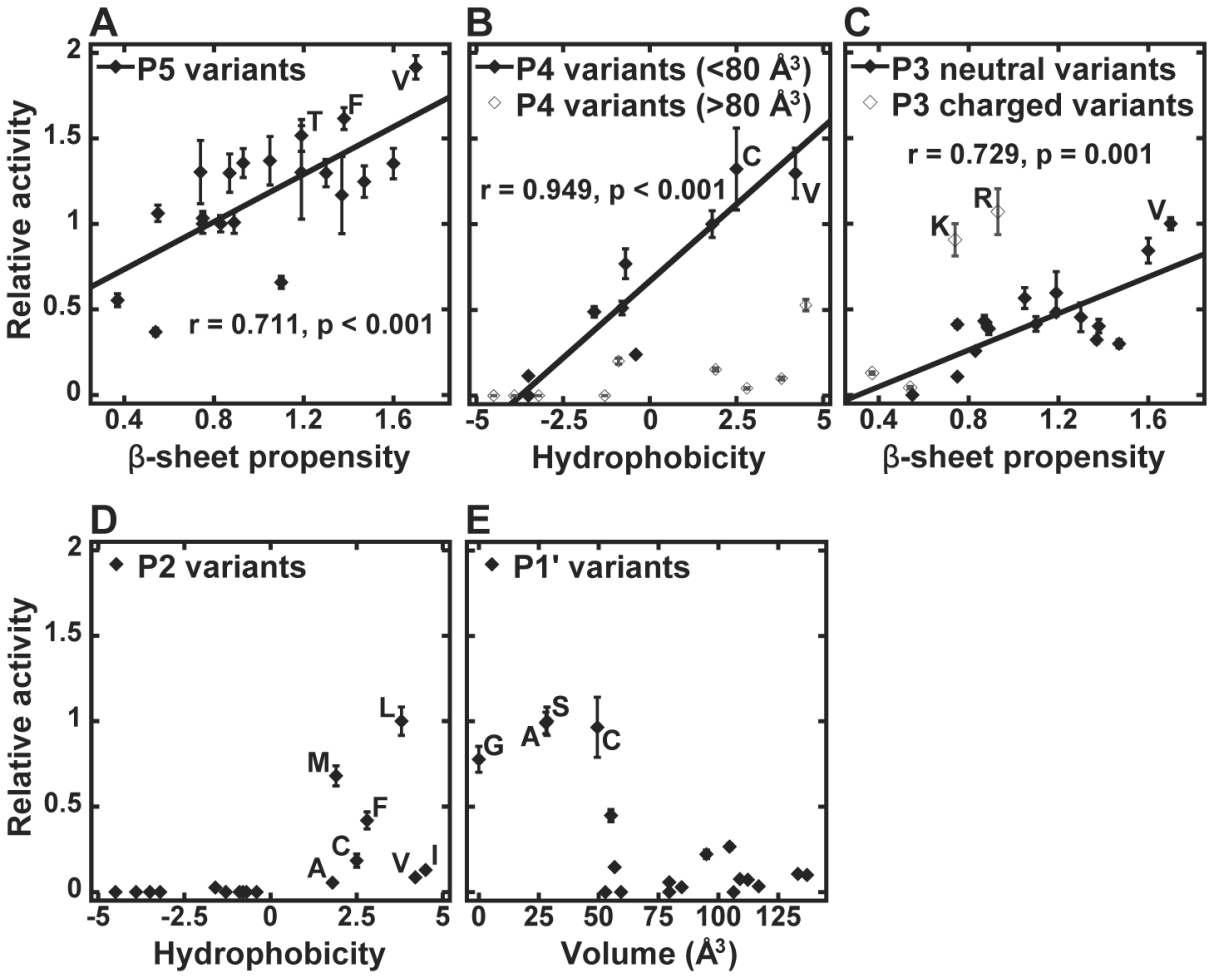
Substrate specificity for SARS-CoV 3CL^pro^. The relative activity significantly correlated with various structural properties of substituting residues. (A) At P5 position, the relative activity correlated well with the β-sheet propensity (r = 0.711, p<0.001). (B) At P4 position, significant correlation was observed for hydrophobicity (r = 0.587, p = 0.008). The correlation was improved (r = 0.942, p<0.001) when only residues with side chain volumes of <80 Å^3^ (Ala, Asn, Asp, Cys, Glu, Gly, Pro, Ser, Thr and Val) were included. (C) The relative activity on P3 variants were correlated with β-sheet propensity (r = 0.510, p = 0.022). Increase in the correlation (r = 0.729, p = 0.001) was found after neglecting charged residues (Arg, Asp, Glu and Lys). (D) Only variants with hydrophobic residues (Ala, Cys, Ile, Leu, Met, Phe, and Val) at P2 position were cleavable. (E) The relative activity on P1' variants with side chain volume of <50 Å^3^ (Ala, Cys, Gly and Ser) were higher than that on others.

All substitutions at P5 position were cleavable, and the relative activity ranged from 0.37 to 1.92. Many substitutions resulted in activity significantly higher than that for WT substrate (Figure 2). S5V (1.92±0.07) was the most preferred substrate variant, followed by S5F (1.62±0.06) and S5T (1.52±0.09). A strong correlation was observed between the relative activity and β-sheet propensity (r = 0.711, p<0.001) (Table 1, Figure 3A). The relative activity also correlated well with the hydrophobicity of substituting residues (r = 0.573, p = 0.008) (Table 1).

### P4 position prefers small hydrophobic residues

The best substitutions were Cys and Val, with relative activity of 1.32±0.24 and 1.30±0.15, respectively (Figure 2). The relative activity correlated well with hydrophobicity (r = 0.587, p = 0.006) (Table 1). The correlation was more evident (r = 0.942, p<0.001) when we excluded residues with side chain volume >80 Å^3^ from the analysis (Figure 3B). From the crystal structure of 3CL^pro^-substrate complex, the side chain of P4 is completely buried inside a small hydrophobic pocket [23]. Our data suggest that for those residues that are small enough to fit into the binding pocket, the relative activity is directly proportional to the hydrophobicity of the substituting residues. No observable cleavage was detected for charged residues (Arg, Asp, Glu and Lys), probably due to the high desolvation penalty for burial of charges inside the hydrophobic pocket.

### P3 position prefers residues with high β-sheet propensity

The relative activity for P3 variants correlated well with β-sheet propensity (r = 0.510, p = 0.022) (Table 1). As discussed above, P3 position favors positively charged residues over negatively charged one. After excluding the charged residues, we found that 3CL^pro^ activity was directly proportional to the β-sheet propensity of substituting residues (r = 0.729, p = 0.001) (Figure 3C). In the crystal structure of 3CL^pro^-substrate complex, the P3-Val is in β-sheet conformation, which facilitates the formation of two hydrogen bonds between backbone peptide groups of P3-Val and Glu166 of 3CL^pro^ [23]. Residues with high β-sheet propensity at P3 position may help to maintain these two hydrogen bonds and results in higher protease activity.

### P2 position prefers hydrophobic residues without β-branch

Detectable cleavage was only observed for hydrophobic substitutions at P2 position (Figure 2, 3D). When all 20 residues were included in the correlation analysis, the relative activity was found to correlate with hydrophobicity (r = 0.590, p = 0.006) (Table 1). The most favorite residue at P2 position was Leu (1.00±0.08), followed by Met (0.68±0.06) and Phe (0.42±0.05). On the other hand, β-branched residues like Ile (0.13±0.01) and Val (0.09±0.01) were less preferred, although their hydrophobicity is similar to that of Leu. Taken together, our results suggest that P2 position prefers hydrophobic residues without β-branch.

### P1 position tolerates His and Met

The substrate was cleavable when P1 position was a Gln, His or Met (Figure 2). Other substitutions were not cleavable. The most favorable residue was Gln, which is an invariant residue at P1 position of the 3CL^pro^ substrate sequences. Substitution to His or Met resulted in reduced relative activities of 0.26±0.02 and 0.10±0.01, respectively. Our observation that P1-His was cleavable is consistent with another study by Goetz *et al.* based on tetrapeptide substrates [19]. However, in their case, the activity of P1-His substrate was even higher than that of the WT sequence of P1-Gln.

**Figure 4:**
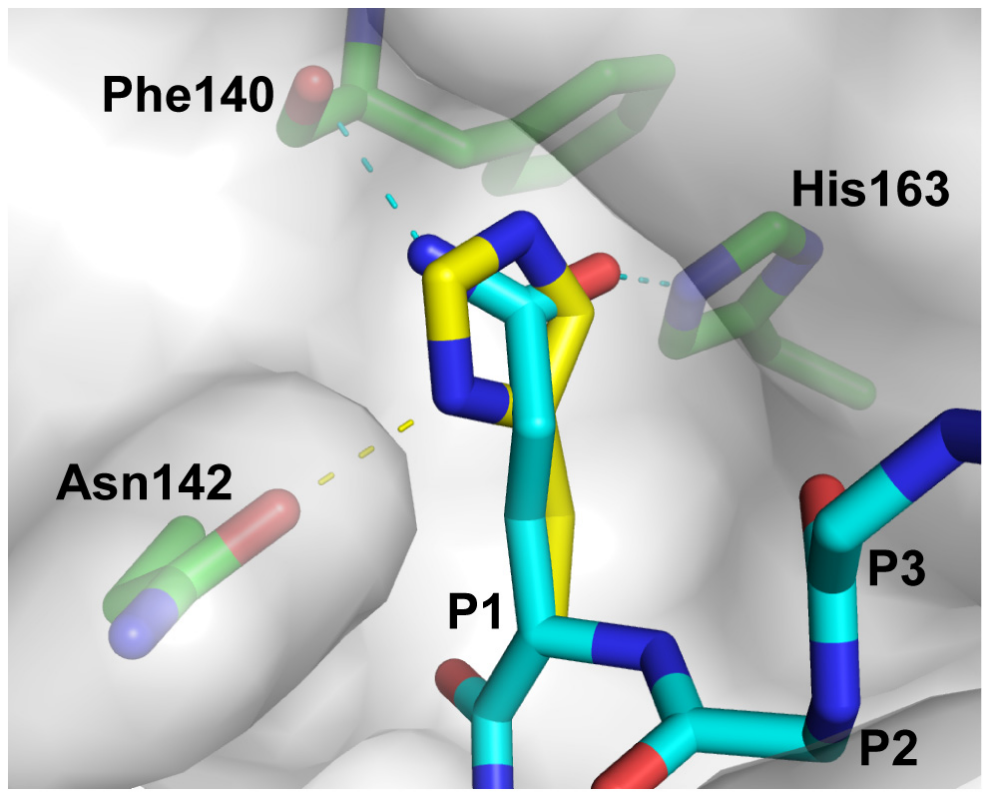
Modeling how 3CL^pro^ recognizes P1-His. In the 3CL^pro^-substrate complex (PDB: 2Q6G), amide group of P1-Gln of the WT substrate sequence (thin stick) forms hydrogen-bonds with the Nε_2_ atom of His163 and the backbone carbonyl group of Phe140. P1-Gln was substituted to His (thick stick) *in silico* using the program SWISS-PDBViewer [24]. The rotamer of P1-His was selected to avoid steric hindrance and to optimize for hydrogen bond formation. The modeled structure was then energy minimized using a GROMOS force-field implemented in SWISS-PDBViewer. It was found that P1-His can fit into the substrate binding pocket and form hydrogen bond to the amide group of Asn142.

In the crystal structure of 3CL^pro^-substrate complex, the Oε_1_ and Nε_2_ atoms of P1-Gln form hydrogen-bonds to Nε_2_ atom of His163 and backbone carbonyl group of Phe140, respectively (Figure 4). We modeled how 3CL^pro^ recognizes P1-His using SWISS-PDBViewer [24]. In the modeled structure, although P1-His can fit into substrate binding pocket without steric hindrance, it is no longer in an optimal position to form hydrogen bonds with His163 and Phe140 (Figure 4). Instead, the Nε_2_ atom of P1-His position can form a hydrogen bond with the amide group of Asn142. From this point of view, substitution of His at P1 position should weaken the enzyme-substrate interaction, which justified our observation that the P1-His is a poorer substrate than P1-Gln.

### P1' prefers small residues

The relative activity of P1' variants negatively correlated with the side chain volume (r = −0.660, p = 0.002) (Table 1). The most preferred residues at P1' position were Ser (1.00±0.08), Ala (0.99±0.06), Cys (0.97±0.18), and Gly (0.78±0.08). Substitutions with residues larger than Cys resulted in dramatic decreases in the relative activity (Figure 3E). Our results suggest that P1' position prefers small residues with side chain volumes less than 50 Å^3^.

### P2' and P3' positions have no strong preference

No significant correlation was found for P2' and P3' positions except the preference for positively charged residues discussed above (Table 1). However, it was noted that small residues such as Gly, Ala and Ser tend to have higher relative activity than the other large residues at P2' position.

### Combining preferred residues generate ‘Super-active’ substrate sequences

**Figure 5:**
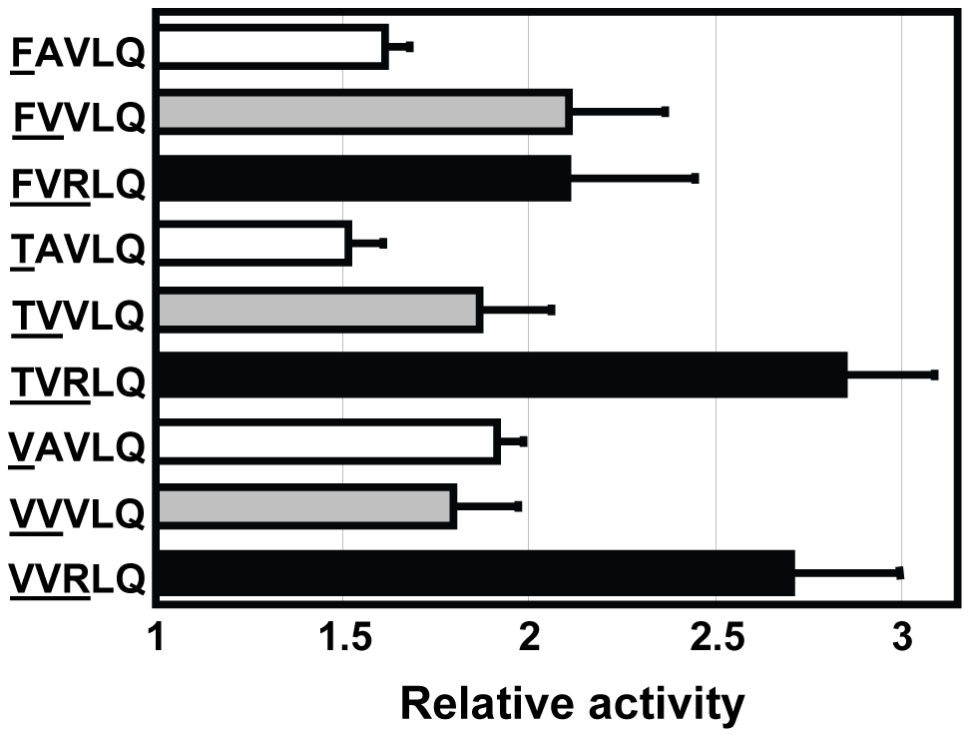
Super-active substrates were created by combining the best residues at P5 to P1 positions. Three variants with double-substitution (grey bar) and three variants with triple-substitution (solid bar) were created, and their relative activities were measured. The relative activities of FVVLQ↓SGF, TVVLQ↓SGF, VVVLQ↓SGF, FVRLQ↓SGF, TVRLQ↓SGF and VVRLQ↓SGF were 2.11±0.26, 1.87±0.19, 1.80±0.17, 2.10±0.34, 2.84±0.25 and 2.71±0.29, respectively.

Our results showed that substitutions to Phe, Thr and Val at P5 position and to Val at P4 position resulted in significant increases in 3CL^pro^ activity (Figure 2). We also showed that P3 position favors positively charged residues. To test if we can generate a ‘super-active’ substrate sequence by combining the best substitutions at these positions, we created three variants with double-substitution (FVVLQ↓SGF, TVVLQ↓SGF and VVVLQ↓SGF) and three variants with triple-substitution (FVRLQ↓SGF, TVRLQ↓SGF and VVRLQ↓SGF). The relative activity of 3CL^pro^ against these substrate sequences was determined (Figure 5). In general, the relative activity was further increased by introduction of more favorable substitutions. Triple substitution resulted in the best substrate sequence, TVRLQ↓SGF, with a relative activity of 2.84±0.25. Noteworthy, docking simulation by Phakthanakanok *et al.* ranked TVKLQ↓AGF and TVRLQ↓AGF as the sequences with the lowest docking energy for 3CL^pro^-substrate interaction [25].

### Comparison with previous studies on the substrate specificity of 3CL^pro^

Fan *et al.* introduced a few selected single-substitutions at P5 to P1' positions to an octapeptide substrate, and monitored the 3CL^pro^ activity by high performance liquid chromatography [16]. In general, the protease activity measured using their 28 octapeptide substrate variants agreed with the profile reported in our study. For example, both studies showed that substitutions at P5 position resulted in substrate variants with activity higher than that for WT, suggesting that P5 residue plays an important role in the 3CL^pro^-substrate interaction. Consistent with our suggestion that positively charged residues are preferred at P3 position, Fan *et al.* showed that the P3-Lys substrate variant also had a relative activity higher than the WT P3-Val substrate.

Goetz *et al.* used a fully degenerate tetrapeptide library to study the substrate specificity of 3CL^pro^ at P4 to P1 positions [19]. The library consisted of 20
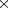
4 sub-libraries, each consisted of a mixture of 20^3^ tetrapeptides with one common residue at a particular position and degenerate residues at the other positions. Consistent with our results, they showed that 3CL^pro^ can cleave both His and Gln at P1 position. It is noteworthy that the reported protease activity for P1-His substrates was slightly higher than that for the P1-Gln substrates. In contrast, our data suggested that P1-His is cleavable but with a lower relative activity of 0.26±0.02. Goetz *et al.* argued that the binding mode of His and Gln to the S1 pocket is similar because the Nε_2_ and Nε_1_ atoms of P1-His can take the approximate positions of Nε_2_ and Oε_1_ atoms of P1-Gln. However, a close inspection of their models revealed that such binding mode of P1-His requires structural changes of the backbone atoms of the P1 residue. The structural changes may be accommodated in their tetrapeptide substrates, which lack residues beyond P1' position that may restrict the backbone conformation of the substrate. In contrast, we argue that for the protein substrate used in our study, it is likely that the backbone conformation of the substrate will be held by extensive interaction of residues from P5 to P3' positions. As discussed above, our model suggests that P1-His should form weaker interaction with the 3CL^pro^ (Figure 4), justifying the observation that P1-Gln is preferred over P1-His in the native cleavage sequences in the SARS-CoV polyproteins.

Moreover, the results of Goetz *et al.* indicated that substrates containing P2-Phe was not cleavable [19]. This finding is in direct contradiction with the results reported in this study and in the study of Fan *et al.* [16], and with the fact that P2-Phe is naturally occurring in the C-terminal autocleavage sequence of 3CL^pro^. Considering that the protease activity measured in the study of Goetz *et al.* represented the ensemble average of a mixture of 20^3^ degenerate peptides, we speculate that their results could be biased by many non-cleavable combinations of sequences within their libraries.

### Concluding remarks

In this study, the substrate specificity of 3CL^pro^ was profiled using a library of protein substrates. The effect of residue substitution at P5 to P3' positions were investigated (Table 2). The comprehensive data obtained allowed us to quantitatively correlate the substrate specificity in terms of side chain volume, hydrophobicity and secondary structure propensities. Not only our results are consistent with some of the previous observations, novel insights into the substrate specificity were obtained in this study. First, positively charged residues are consistently preferred over negatively charged ones at solvent-exposed positions such as P5, P3, P3'. Second, the 3CL^pro^ activity is directly proportional to hydrophobicity for small residues at P4, and to β-sheet propensities at P5 and P3 positions. Third, residues larger than Cys are not favored at P1' position. Fourth, the most favorite residue at P1 position is Gln, but P1-His and P1-Met are also cleavable. Our results suggest the existence of a strong structure-activity relationship between 3CL^pro^ and its substrates. The substrate specificity profiled in this study can be used as a benchmark for better computational simulation for 3CL^pro^-substrate/inhibitor interaction, and may provide a guideline for a rational based design of potent inhibitors.

**Table 2:** Summary of SARS-CoV 3CL^pro^ substrate specificity at P5 to P3' positions.

Position	WT residue	Major specificity	The most preferred residue (relative activity ± SD)
P5	Ser	Residues with high β-sheet propensity	Val (1.92±0.07); Phe (1.62±0.06); Thr (1.52±0.09)
P4	Ala	Small hydrophobic residues	Cys (1.32±0.24); Val (1.30±0.15)
P3	Val	Positively charged residues; Residues with high β-sheet propensity	Arg (1.07±0.13); Val (1.00±0.04)
P2	Leu	Hydrophobic residues without β-branch	Leu (1.00±0.08)
P1	Gln	Gln	Gln (1.00±0.08)
‘P1’	Ser	Small residues with side chain <50 Å^3^	Ser (1.00±0.08); Ala (0.99±0.06); Cys (0.97±0.18)
‘P2’	Gly	No strong preference	Ser (1.29±0.12)
‘P3’	Phe	No strong preference	Arg (1.10±0.03)

## Materials and Methods

### Production of SARS-CoV 3CL^pro^

DNA fragment encoding the protease was amplified by polymerase chain reaction based on strain CUHK-Su10 sequence (GenBank AY282752) [26]. The coding sequence of 3CL^pro^ was cloned into a fusion-protein expression vector so that the 3CL^pro^ is tagged with poly-Histidine-maltose binding protein (His_6_-MBP) at the N-terminus. A factor Xa cleavage sequence was present between 3CL^pro^ and the tag for subsequent removal of the tag by factor Xa digestion. Expression of recombinant 3CL^pro^ was induced by 0.4 mM of isopropyl β-D-1-thiogalactopyranoside in *E. coli* strain BL21 (DE3) pLysS during mid-log phase. The cells were grown at 37°C for 4 hours, followed by sonication in buffer A (20 mM Tris, 20 mM NaCl, pH 7.8) with 10mM imidazole. Soluble fraction was subjected to immobilized metal ion affinity chromatography (IMAC), and the recombinant protein was eluted by buffer A with a gradient of 10 to 300 mM imidazole. The His_6_-MBP tag was removed by factor Xa digestion in 20 mM Tris, 50 mM NaCl, 2 mM CaCl_2_, pH 7.4 overnight, and by IMAC. The protease was finally purified by G75 size exclusion column pre-equilibrated with buffer A. Elution profile showed that the protease was a dimer in solution.

### Production of protein substrate library

A pET3a plasmid encoding the recombinant WT substrate (His_6_-CFP-TSAVLQSGFRKM-YFP) was constructed (Figure 1A). For construction of the protein substrate library, 19×8 mutations were introduced at P5 to P3' positions by the QuikChange mutagenesis kit (Stratagene). The protein substrate expression was induced by 0.1 mM isopropyl β-D-1-thiogalactopyranoside, followed by shaking of culture at 22°C overnight. After sonication, the expressed protein was purified by IMAC and stored in buffer A.

### FRET assay for 3CL^pro^ proteolytic rate measurement

35 µM of the recombinant substrate was rapidly mixed with 1 to 4 µM of 3CL^pro^ in 96-well black Optiplate. The cleavage of the protein substrate was monitored by FRET using EnVision 2101 Multilabel Plate Reader. The reaction mixture was excited by light passing though a 430 nm filter (with 8 nm bandwidth), and the intensity of emitted fluorescence passing though a 530 nm filter (with 10 nm bandwidth) was recorded. For Cys variants, 2.5 mM tris(2-carboxyethyl)phosphine was added to prevent disulphide bond formation.

The observed rate constant, k_obs_, was obtained by fitting the emitted fluorescence at 530 nm to a single exponential decay. The specific activity of 3CL^pro^ on variant substrates, A_VAR_, was determined by the slope of k_obs_/[3CL^pro^], and was normalized against the value for WT sequence, A_WT_, to obtain the relative activity:
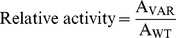
The assay for each substrate was performed in triplicate.

### Correlation with structural properties

The relative activity was correlated with various structural properties of substituting residues, including side chain volume [20], hydrophobicity [21], and α-helix and β-sheet propensities [22] (Table S2). Coefficients and p-values of the correlations were obtained.

## Supporting Information

Table S1 SARS-CoV 3CL^pro^ relative activity on the substrate variants. ‘ND’ stands for non-detectable cleavage.
(0.07 MB DOC)

Table S2 Scales for quantification of structural properties. The side chain volume was derived from the partial molar volume of amino acids reported in Lee *et al.* [20]. Scales of hydrophobicity and secondary structure propensities were obtained from Kyte & Doolittle [21] and Chou & Fasman [22], respectively.
(0.05 MB DOC)
