# Supplementary material for: Profiling of Substrate Specificity of SARS-CoV 3CLpro
Source: PLoS One. 2010 Oct 6;5(10):e13197. doi: 10.1371/journal.pone.0013197 (PMC2950840; doi:10.1371/journal.pone.0013197)
Supplement: Table S1 — SARS-CoV 3CLpro relative activity on the substrate variants. ‘ND’ stands for non-detectable cleavage. (0.07 MB DOC) [file pone.0013197.s001.doc]

**Table S1.** SARS-CoV 3CL^pro^ relative activity on the substrate variants. ‘ND’ stands for non-detectable cleavage.

| **Residue** | **Position at the autocleavage sequence (WT residue)** | | | | | | | |
| --- | --- | --- | --- | --- | --- | --- | --- | --- |
|  | **P5 (Ser)** | **P4 (Ala)** | **P3 (Val)** | **P2 (Leu)** | **P1 (Gln)** | **P1’ (Ser)** | **P2’ (Gly)** | **P3’ (Phe)** |
| A | 1.00 ± 0.05 | 1.00 ± 0.08 | 0.26 ± 0.01 | 0.06 ± 0.01 | ND | 0.99 ± 0.06 | 1.05 ± 0.19 | 0.56 ± 0.03 |
| C | 1.30 ± 0.27 | 1.32 ± 0.24 | 0.60 ± 0.12 | 0.18 ± 0.04 | ND | 0.97 ± 0.18 | 0.69 ± 0.12 | 0.83 ± 0.19 |
| D | 0.37 ± 0.02 | ND | 0.04 ± 0.01 | ND | ND | ND | 0.28 ± 0.02 | 0.26 ± 0.02 |
| E | 0.55 ± 0.04 | ND | 0.13 ± 0.01 | ND | ND | ND | 0.23 ± 0.02 | 0.42 ± 0.04 |
| F | 1.62 ± 0.06 | 0.04 ± 0.00 | 0.40 ± 0.04 | 0.42 ± 0.05 | ND | 0.11 ± 0.01 | 0.66 ± 0.04 | 1.00 ± 0.03 |
| G | 1.03 ± 0.04 | 0.24 ± 0.02 | 0.11 ± 0.01 | ND | ND | 0.78 ± 0.08 | 1.00 ± 0.05 | 0.35 ± 0.01 |
| H | 1.30 ± 0.11 | ND | 0.43 ± 0.03 | ND | 0.26 ± 0.02 | 0.22 ± 0.02 | 0.62 ± 0.06 | 0.84 ± 0.07 |
| I | 1.35 ± 0.09 | 0.53 ± 0.03 | 0.84 ± 0.07 | 0.13 ± 0.01 | ND | ND | 0.37 ± 0.06 | 0.81 ± 0.08 |
| K | 1.30 ± 0.19 | ND | 0.91 ± 0.09 | ND | ND | 0.03 ± 0.01 | 0.80 ± 0.05 | 0.94 ± 0.13 |
| L | 1.30 ± 0.08 | 0.10 ± 0.01 | 0.45 ± 0.08 | 1.00 ± 0.08 | ND | 0.08 ± 0.01 | 0.44 ± 0.04 | 0.64 ± 0.02 |
| M | 1.37 ± 0.14 | 0.15 ± 0.01 | 0.57 ± 0.06 | 0.68 ± 0.06 | 0.10 ± 0.01 | 0.27 ± 0.02 | 0.56 ± 0.02 | 0.61 ± 0.04 |
| N | 1.01 ± 0.06 | 0.11 ± 0.01 | 0.39 ± 0.03 | ND | ND | 0.15 ± 0.01 | 0.60 ± 0.05 | 0.62 ± 0.02 |
| P | 1.06 ± 0.05 | 0.49 ± 0.03 | ND | 0.03 ± 0.00 | ND | ND | ND | 0.43 ± 0.02 |
| Q | 0.66 ± 0.04 | ND | 0.41 ± 0.04 | ND | 1.00 ± 0.08 | 0.03 ± 0.00 | 0.81 ± 0.06 | 0.59 ± 0.05 |
| R | 1.36 ± 0.08 | ND | 1.07 ± 0.13 | ND | ND | 0.07 ± 0.01 | 0.79 ± 0.05 | 1.10 ± 0.03 |
| S | 1.00 ± 0.05 | 0.51 ± 0.04 | 0.41 ± 0.02 | ND | ND | 1.00 ± 0.08 | 1.29 ± 0.12 | 0.72 ± 0.07 |
| T | 1.52 ± 0.09 | 0.77 ± 0.09 | 0.48 ± 0.02 | ND | ND | 0.45 ± 0.04 | 0.71 ± 0.12 | 0.82 ± 0.02 |
| V | 1.92 ± 0.07 | 1.30 ± 0.15 | 1.00 ± 0.04 | 0.09 ± 0.01 | ND | 0.06 ± 0.01 | 0.67 ± 0.06 | 0.70 ± 0.03 |
| W | 1.17 ± 0.22 | 0.20 ± 0.02 | 0.32 ± 0.02 | ND | ND | 0.08 ± 0.01 | 0.40 ± 0.03 | 0.61 ± 0.02 |
| Y | 1.25 ± 0.09 | ND | 0.30 ± 0.02 | ND | ND | 0.10 ± 0.01 | 0.64 ± 0.03 | 0.85 ± 0.06 |
