# Supplementary material for: Profiling of Substrate Specificity of SARS-CoV 3CLpro
Source: PLoS One. 2010 Oct 6;5(10):e13197. doi: 10.1371/journal.pone.0013197 (PMC2950840; doi:10.1371/journal.pone.0013197)
Supplement: Table S2 — Scales for quantification of structural properties. The side chain volume was derived from the partial molar volume of amino acids reported in Lee et al. [20]. Scales of hydrophobicity and secondary structure propensities were obtained from Kyte & Doolittle [21] and Chou & Fasman [22], respectively. (0.05 MB DOC) [file pone.0013197.s002.doc]

**Table S2.** Scales for quantification of structural properties. The side chain volume was derived from the partial molar volume of amino acids reported in Lee *et al.* [20]. Scales of hydrophobicity and secondary structure propensities were obtained from Kyte & Doolittle [21] and Chou & Fasman [22], respectively.

| **Residue** | **Side chain volume (Å^3^)** | **Hydrophobicity** | **α-helix propensity** | **β-sheet propensity** |
| --- | --- | --- | --- | --- |
| A | 28.0 | 1.8 | 1.42 | 0.83 |
| C | 49.5 | 2.5 | 0.70 | 1.19 |
| D | 52.8 | -3.5- | 1.01 | 0.54 |
| E | 79.5 | -3.5- | 1.51 | 0.37 |
| F | 133.2 | 2.8 | 1.13 | 1.38 |
| G | 0 | -0.4- | 0.57 | 0.75 |
| H | 95.0 | -3.2- | 1.00 | 0.87 |
| I | 106.3 | 4.5 | 1.08 | 1.60 |
| K | 116.8 | -3.9- | 1.16 | 0.74 |
| L | 109.0 | 3.8 | 1.21 | 1.30 |
| M | 104.7 | 1.9 | 1.45 | 1.05 |
| N | 56.7 | -3.5- | 0.67 | 0.89 |
| P | 59.3 | -1.6- | 0.57 | 0.55 |
| Q | 84.7 | -3.5- | 1.11 | 1.10 |
| R | 112.3 | -4.5- | 0.98 | 0.93 |
| S | 28.5 | -0.8- | 0.77 | 0.75 |
| T | 55.2 | -0.7- | 0.83 | 1.19 |
| V | 79.5 | 4.2 | 1.06 | 1.70 |
| W | 170.2 | -0.9- | 1.08 | 1.37 |
| Y | 137.0 | -1.3- | 0.69 | 1.47 |
